# The CIMMYT Australia ICARDA Germplasm Evaluation concept: a model for international cooperation and impact

**DOI:** 10.3389/fpls.2024.1435837

**Published:** 2024-07-30

**Authors:** Richard M. Trethowan, Julie M. Nicol, Amit Singh, Ravi P. Singh, Wuletaw Tadesse, Velu Govidan, Leonardo Crespo-Herrera, Brian Cullis, Luke Mazur, Mark Dieters, Sandra Micallef, Terence Farrell, Robin Wilson, Ky Mathews

**Affiliations:** ^1^ The Plant Breeding Institute, Sydney Institute of Agriculture, The University of Sydney, Cobbitty, NSW, Australia; ^2^ International Maize and Wheat Improvement Centre (CIMMYT), Texcoco, Mexico; ^3^ International Centre for Agricultural Research for the Dry Areas (ICARDA), Station Exp. INRA, Rabat, Morocco; ^4^ Centre for Biometrics and Data Science for Sustainable Primary Industries, National Institute for Applied Statistics Research Australia, University of Wollongong, Wollongong, NSW, Australia; ^5^ School of Agriculture and Food Sciences, University of Queensland, St. Lucia, QLD, Australia; ^6^ Grains Research and Development Corporation, Barton, ACT, Australia; ^7^ Integrated Breeding Platform, Perth, Australia

**Keywords:** wheat, CIMMYT Australia ICARDA Germplasm Evaluation, genetic diversity, impact, benefit-cost

## Abstract

Bread wheat germplasm is accessed from the International Maize and Wheat Improvement Centre (CIMMYT) and the International Centre for Agricultural Research in the Dry Areas (ICARDA) by Australian wheat breeders and researchers through the CIMMYT Australia ICARDA Germplasm Evaluation (CAIGE) program. The CAIGE program coordinates the selection, importation, quarantine, dissemination, and evaluation of the imported bread wheat germplasm and the management of associated data and information. This paper describes the CAIGE model and assesses both the genetic and economic impacts of these materials on the Australian wheat industry after commercialisation of wheat breeding in the early 21st century and the establishment of CAIGE. The CAIGE concept was validated using data collected and analysed from multi-environment trials between 2017 and 2020. The impact of cultivars with and without CAIGE contribution to pedigree on yield was estimated using production-by-variety statistics. Net gain in yield, estimated as the yield difference between CAIGE and Non-CAIGE varieties, was multiplied by the percentage contribution to pedigree to estimate the additional yield. The CAIGE bread wheat program identified diverse, high-yielding, and disease-resistant germplasm and significantly improved the capture and dissemination of information. The benefit-cost ratio, calculated as the sum of benefits divided by investments, indicated that, for every dollar invested in CAIGE, a further $20 was generated in benefits. The internal rate of return was estimated at 163% and the modified rate at 18%. The benefits of these international materials to Australian wheat breeding remained significant.

## 1 Introduction

The wheat breeding programs of the Consultative Group on International Agricultural Research (CGIAR) have contributed to significant improvements in wheat productivity globally (Pardey, 2011). Materials developed by the CGIAR centres, specifically the International Maize and Wheat Improvement Centre (CIMMYT) and the International Centre for Agricultural Research in the Dry Areas (ICARDA), are deployed in adaptation-based nurseries globally, and local breeders and researchers select materials on the basis of performance and available information. These materials are targeted to developing countries, and adoption rates have climbed steadily since the inception of the Green Revolution. Currently, 92% of all wheat cultivars sown in the developing world either are direct releases of CGIAR materials or have CGIAR parents (Lantican et al., 2016).

The bread wheat germplasm of the CGIAR also has had a significant impact in Australia (Brennan and Quade, 2006), despite Australia not being a CGIAR target country, quarantine limitations to importing trial quantities of seed and the *ad hoc* assessment of the germplasm after quarantine.

### 1.1 A new approach to exploiting imported CGIAR diversity

The impact of CGIAR wheat germplasm in Australia was significant despite the shortcomings in germplasm acquisition and evaluation noted earlier. Meng and Brennan (2009) provide a historical prospective of wheat breeding programs in Australia and their association with CIMMYT and ICARDA programs. The CIMMYT Australia ICARDA Germplasm Evaluation (CAIGE) program was established in 2007 to better coordinate the identification, importation, quarantine, distribution, and evaluation of wheat germplasm initially from CIMMYT and from 2013 onwards, materials from ICARDA were included (http://www.caigeproject.org.au). The aim of CAIGE was to derive maximum benefit from the international investment in public wheat breeding for the benefit of Australian grain growers. Prior to the formal establishment of CAIGE, germplasm from these centres was introduced on an *ad hoc* basis, and no supporting data or information on local adaptation was available to wheat breeders and researchers.

Nevertheless, it is anticipated that this better coordinated strategy has improved the local uptake and use of bread wheat germplasm from the CGIAR.

### 1.2 The economic impact of CGIAR bread wheat germplasm

Estimates of the international impact of the CGIAR investment in wheat breeding in terms of increased production range from benefit-cost ratios of 16:1 for the period 1966–1997 (Heisey et al., 2002) to more than 100:1 (Lantican et al., 2016) between 1994 and 2014.

While the CGIAR wheat germplasm is targeted to developing countries, there are significant spill over benefits to Australia, although these are offset by reduced wheat prices, a consequence of the success of the CGIAR in increasing global productivity (Brennan and Quade, 2006). A previous economic impact analysis estimated the net annual benefit of the CGIAR germplasm introduced to Australia between 1973 and 2001 at $30.3 million in 2003 AUD dollars (Brennan and Quade, 2004). This equated to a benefit-cost ratio of 30:1, as Australia committed around $AUD1m annually to CIMMYT at the time. The Brennan and Quade analysis covered the period of the Green Revolution including the introduction of the first semi-dwarf cultivars that significantly increased wheat productivity globally (Trethowan et al., 2007). Wheat breeding was commercialised at the turn of the century in Australia, and, with few exceptions, CGIAR materials were used as parents only (Brennan and Quade, 2006), primarily because the international materials did not generally meet Australia’s strict grain quality classifications.

The Brennan and Quade (2004) analysis identified commercial wheat varieties derived from CIMMYT materials only; the bread wheat breeding program at ICARDA was managed by CIMMYT at this time and did not represent a demarcation of materials. These authors used a database of Australian crop production by cultivar statistics to calculate the tonnes produced by CIMMYT-derived cultivars each year in comparison to those without a CIMMYT parentage. The difference between the yields of CIMMYT- and non-CIMMYT–derived cultivars indicated the extra production attributable to CIMMYT. This extra annual production was multiplied by the average annual price to derive a dollar value for each year. The estimated benefit values were subtracted from the current value of the sums invested over the life of the program to derive the net present value of the investment, the benefit-cost ratio, and the internal rate of return.

The analysis by Brennan and Quade (2004) calculated a value for the yield advantage of wheat cultivars carrying CIMMYT diversity versus yield gains from cultivars where materials did not have CIMMYT parentage. They compared the full yield advantage of CIMMYT-derived cultivars relative to non-CIMMYT cultivars. However, most CIMMYT and ICARDA germplasm is used as parents in Australia, and the partial contribution to cultivar pedigree and, therefore, yield improvement was not considered.

This paper describes the CAIGE collaborative model and aims to assess the genetic and economic impacts of these materials on the Australian wheat industry after commercialisation of wheat breeding and the establishment of CAIGE. The Brennan and Quade (2004) approach for assessing economic impact was modified to include percentage pedigree contribution of CGIAR germplasm to recent Australian cultivars.

## 2 Materials and methods

### 2.1 The CAIGE concept and structure

The CAIGE concept, jointly funded by the Grains Research and Development Corporation (GRDC) and the Australian crop breeding community, was initiated in 2007 and has been modified and improved over the years. While bread wheat, durum wheat, and barley germplasm and associated information from CIMMYT and ICARDA is accessed by Australian wheat scientists and wheat breeders through the CAIGE program, only bread wheat is described in this paper. Nevertheless, the same principles apply to both durum wheat and barley.

Stage 1. Germplasm selection, importation, and quarantine

Approximately 300 bread wheat lines from CIMMYT or ICARDA are selected each year. Representatives of Australian wheat breeding companies visit CIMMYT’s major field breeding site at Ciudad Obregon in Mexico or ICARDA’s Marchouch field site in Morocco in alternating years. The lines are selected primarily on plant height and maturity, following which the set is reduced using grain colour, grain yield, disease, and industrial quality information provided by the CGIAR breeders. While the lines are being multiplied at a location with low to no disease expression prior to dispatch to Australia, they are, since 2022, concurrently screened in a global phenotyping platform for traits specifically identified by Australian breeders. The aim is to ensure that at least 20% of the imported set carries diversity for these nominated traits and the information is used to further reduce the set to 250 lines prior to quarantine.

The lines are imported with all accompanying passport data including yield, disease resistance, quality, linked molecular markers, and other genotypic data provided by the respective CGIAR centres. The lines entering Australia are quarantined at the Australian Grains Genebank (AGG). From 2023, all lines in quarantine are genotyped by AGG using a 40K single nucleotide polymorphism (SNP) platform.

Stage 2. Post-quarantine multiplication and concurrent rust testing

Post-quarantine multiplication subsequently takes place at the University of Sydney’s Plant Breeding Institute (PBI) at Narrabri, New South Wales, with concurrent rust disease screening (leaf, stem, and yellow) at Cobbitty, southwest of Sydney, under inoculated field conditions. After harvest, the set is further reduced using the Australian rust data and any additional phenotypic information, including data from global phenotyping platforms provided by the CGIAR centres.

Stage 3. National yield and disease evaluation

Each year, a new cohort of lines is evaluated at up to 14 locations nationally in an optimised multi-environment trial (MET). These sites are sown primarily by collaborating wheat breeding companies, and the data are shared within the CAIGE community. Trial locations are determined by each participating company to represent the three Australian grain cropping zones: north, south, and west (https://grdc.com.au/about/our-industry/growing-regions). Up to 14 Australian released cultivars, nominated by the Australian breeders, are included each year, as well as one to two checks from both CIMMYT and ICARDA.

Stage 4. Data management, dissemination, access, and network coordination

Once the yield and disease data are compiled and analysed, the yield results and individual disease screening data are provided on the CAIGE website (http://www.caigeproject.org.au) with the data stored in a relational database, the Breeding Management System (BMS) (http://www.caigeproject.org.au/breeding-management-system/). Furthermore, the Australian yield results and disease data along with relevant data provided by collaborating CGIAR centres are combined into a single dataset of all lines evaluated in the current year. The analysed data are available through the CAIGE website no later than the end of February each year, thus allowing wheat breeders to make timely sowing decisions.

The CAIGE network comprises many stakeholders, including CGIAR and Australian wheat breeders, wheat researchers, students, grain growers, and other industry personnel. An Annual General Meeting of stakeholders is held at which key results and plans for the next season are discussed and a quarterly newsletter provides stakeholders with updates on progress. The initiative is guided by a steering committee that meets biannually and includes key stakeholders representing public and private Australian organisations, the CGIAR centres, and GRDC. A breeder’s reference group, comprising the core CAIGE team, representatives of each participating commercial company, and the GRDC, oversees the broader strategy.

### 2.2 Genetic impact of the CAIGE program

All data captured on wheat nurseries since the inception of CAIGE are available on the CAIGE website (http://www.caigeproject.org.au). Information includes passport data such as genotype identification number provided by CGIAR centres, Australian Grains Genebank accession numbers, line names and synonyms, pedigree strings in Purdy notation, and selection histories. In this validation, the term “genotype” will be used to represent a wheat breeding line, cultivar, and variety.

To demonstrate the value of the CAIGE model, the bread wheat genotypes evaluated between 2017 and 2020 in Australia were analysed, and comparisons of the performance for CGIAR germplasm with locally bred germplasm were made. A total of 1,321 unique genotypes were evaluated during this period in 34 environments (representing 20 unique locations). The ratio of CIMMYT to ICARDA genotypes varies from year to year with a total annual number ranging between 223 and 501, excluding Australian check genotypes.

The University of Sydney’s field station near Narrabri in the northern grains region was considered the “mother site,” and all genotypes in each annual cohort were evaluated at this site. Roseworthy (managed by Australian Grain Technologies) and Toodyay (managed by EdStar Genetics) represented the southern and western regions, respectively, and were sown in all years, except in 2020 where York replaced Toodyay. All trials, except those at Narrabri, were managed by commercial plant breeding companies and incorporated into their phenotyping pipelines. The data were provided by collaborators in standardised formats for analysis. Data curation to confirm the experimental design integrity and preliminary single trial analyses were performed to identify outliers and calculate trial level reliabilities, as per Mrode (2005), prior to inclusion in the MET analysis. The range of individual trial reliabilities based on the yield analysis ranged from 0.35 to 0.90 (**Table 1**).

**Table 1:** Summary of the CAIGE bread wheat 2017–2020 dataset by year, including ranges for number of environments (trials), number of entries, percentage entry replication, mean yield range for each environment (t/ha), and range in trial reliabilities each year.

Year	Number of locations	Number of lines	Percentage replication (%)	Environment mean yield (t/ha)	Reliability
2017	7	190–234	52–79	1.9–6.6	0.56–0.90
2018	6	194–309	14–70	1.6–5.7	0.53–0.82
2019	7	180–312	18–100	0.6–4.0	0.47–0.85
2020	14	232–501	5–32	1.2–5.6	0.35–0.89

**Figure 1:**
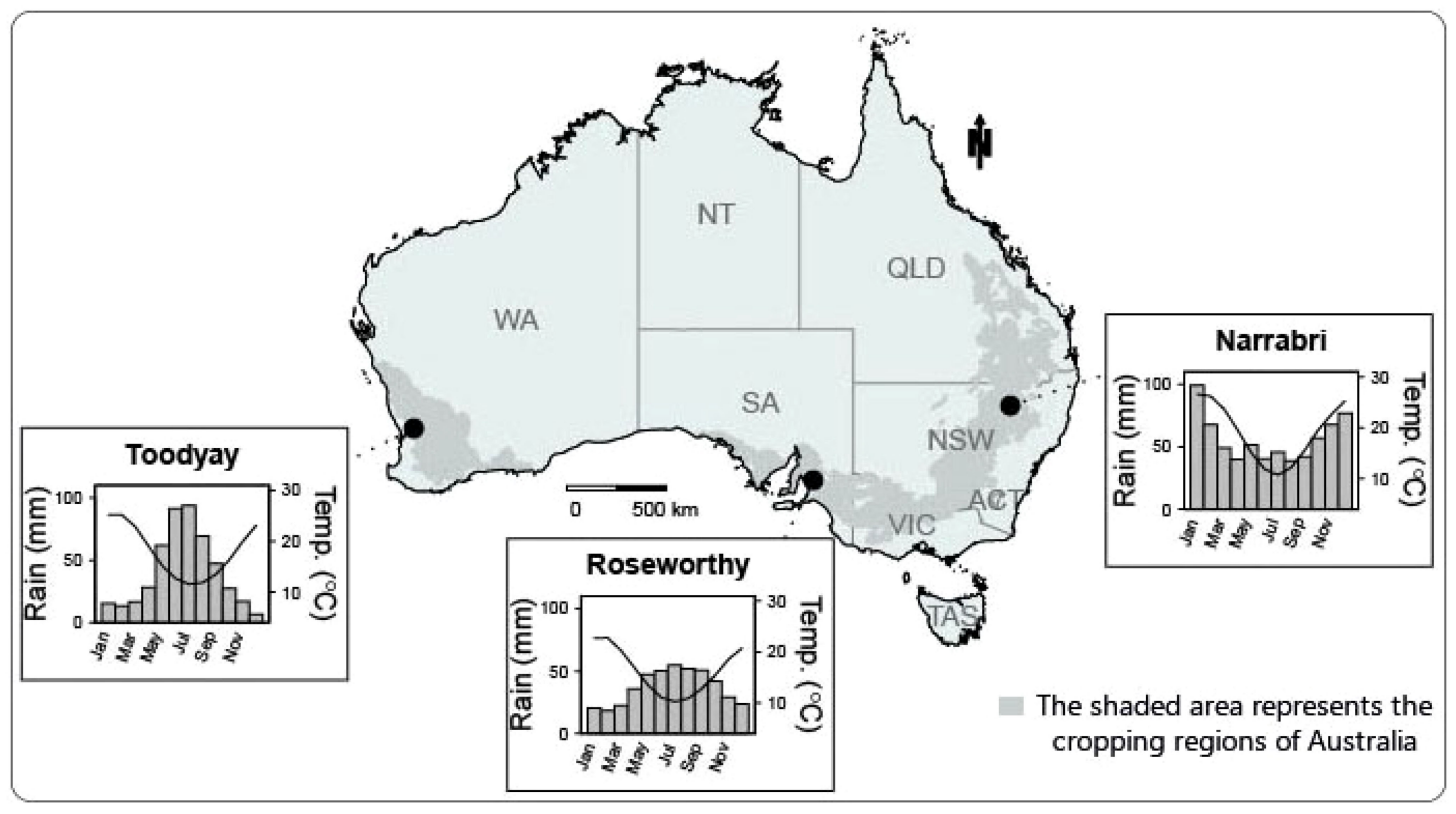
The location of the three key CAIGE trial sites representing the northern, southern, and western cropping zones and their long-term average (1960–2019) rainfall and temperature. Adapted from Chenu et al. (2013).

All trials were sown under rain-fed conditions except Narrabri, where supplementary irrigation was used. The rainfall and temperature patterns for 1960–2020 at the three key sites are illustrated in **Figure 1**. A wide range in mean yield was recorded among environments (**Table 1**).

Quarantine and seed multiplication processes resulted in insufficient seed for all genotypes to be allocated to two plots in each location; hence, near-optimal partially replicated model–based designs, so-called p-rep designs, were generated each year following Cullis et al. (2006) and using the Optimal Design package (odw) (Butler and Cullis, 2023) on the R statistical platform (Kuhn et al. 2010). There were two steps in the design: the allocation of genotype packets to locations and the allocation of genotypes to plots within locations.

To assess genotype-by-environment interaction, genotypes were allocated to as many locations as possible annually. Two packets of each Australian variety were allocated to a location: One packet of each CAIGE genotype was allocated to each location (where possible), and a second packet of each CAIGE genotype was allocated hierarchically to the key locations (Narrabri, Roseworthy, and Toodyay) and all subsequent second packets of CAIGE genotypes were allocated to remaining locations based on maximising collaborator involvement, location constraints such as land availability, and sampling representative growing environments. The resulting MET is unbalanced with respect to genotypes and their within trial replication; however, the imbalance is minimised by ensuring that the maximum number of genotypes are evaluated in the maximum number of locations (environments), thus minimising any potential bias due to over or under-sampling of a target environment.

Individual yield trials were laid out in a rectangular array of rows and columns, with two blocks in either the row or column direction depending on the collaborators’ specifications. Replicated genotypes were allocated across the field layout such that the blocks at each location were near-resolvable. The percent replication (p-rep) ranged from 4.8% at Balaklava 2020 to 100% at Narrabri 2019; 22 trials had a p-rep less than 50% (**Table 1**). In 2020, pedigree information was used to allocate genotypes to locations and plots within locations following the methodology described in Cullis et al. (2020). The combination of many locations (14), large numbers of genotypes (502), and use of pedigree information in the design allowed a lower percent replication than in previous years (5$–24% c.f. greater than 13%).

The terms trial and environment are synonymous and represent a unique year-location combination, except for the 2020 Spring Ridge environment. The Spring Ridge trial was sown in late August 2019 and not considered representative of this region; hence, it was re-evaluated in 2020 alongside the 2020 trial and was therefore co-located. Thus, there are 34 trials in a dataset of 33 environments.

Pedigree information for 90% of genotypes imported from the CGIAR was available in Purdy string notation (Purdy et al., 1968). Pedigree information of the Australian check genotypes was also captured but with less depth. The pedigree information was converted to a numerator relationship matrix, A, following Wright (1922), which was used in a linear mixed model to partition the total genetic effects into additive and non-additive effects (Cullis et al., 2010). The additive genetic effects were equivalent to estimated breeding values and were used for parental selection—a key aim of the CAIGE project. Furthermore, the total genetic effects were generated to assist selection of lines for direct release—should disease and quality traits meet industry standards.

All genotypes were assessed for resistance to key diseases including stripe rust (*Puccinia striiformis* f. sp. tritici), leaf rust (*Puccinia triticina*) and stem rust (*Puccinia graminis*), leaf spot blotch (*Septoria tritici*), Septoria blotch (*S. nodorum*), and the soil- and stubble-borne pathogens tan spot (*Pyrenophora tritici-repentis*) and crown rot (*Fusarium pseudograminearum*). From 2019 onwards, Root Lesion Nematode (*Pratylenchus thornei*) resistance and tolerance screening was performed on a subset of gentoypes, which were selected after yield testing. Septoria nodorum blotch (*Parastagonospora nordorum*) resistance was evaluated by testing host sensitivity to three fungal effectors: ToxA, Tox1, and Tox3.

Disease and yield testing were concurrent, except for the first round of leaf, stem, and stripe rust screening, which occurred after quarantine. Collaborating Australian pathologists scored materials in inoculated fields and/or greenhouses. Data were subsequently converted to a common scale where R is resistant (little or no disease), R-MR is resistant to moderately resistance (trace or disease but a resistant reaction), MR is moderately resistant, MR-MS is moderately resistance to moderately susceptible, MS is moderately susceptible, MS-S is moderately susceptible to susceptible, S is susceptible, and VS is very susceptible.

Data were analysed by the Centre for Bioinformatics and Biometrics at the University of Wollongong, NSW, using a factor analytic linear mixed model (FA-LMM) framework. A key feature of MET datasets is the re-ranking of genotype performance in different environments, known as genotype-by-environment interaction. The FA-LMM parsimoniously models the between environment variance–covariance matrix and usually results in a good fit to the data (Smith et al., 2001; Gogel et al., 2018). FA-LMMs account for the covariances of the GE effects between environments using a small number, k, of (unknown) common factors, which are estimated from the data. Thus the factor analytic model has an order of k (FAk).

The ancestral relationship between genotypes was incorporated into a FA-LMM using the numerator relationship matrix derived from pedigree information (Oakey et al., 2006, Oakey et al., 2007). This enabled the total genotype-by-environment (GE) effects to be partitioned into additive and non-additive GE effects, and their respective between environment genetic variance matrices were modelled with separate FA models (Oakey et al., 2007; Smith and Cullis, 2018). The order, ka or ke, of the respective additive and non-additive FA models increased independently until a good fit to the data was found.

Likelihood ratio tests for nested models, Akaike Information Criterion (AIC) for non-nested models, and descriptive statistics, such as the percentage genetic variance accounted for (%vaf) (Smith et al., 2015), were utilised to determine the model of best fit.

Factor analytic selection tools (FAST) summarise the large amount of information generated from FA-LMM (Smith and Cullis, 2018). These tools measure overall performance (OP), stability [root mean square deviation (RMSD)] and responsiveness for each genotype across all environments in the dataset. OP and RMSD are based on the first factor, which, by design, accounts for the most genetic variance, and responsiveness is based on the remaining k factors. Importantly, all measures are based on the same scale as the data, i.e., t/ha for yield in this dataset.

### 2.3 Economic impact of the CAIGE program

A similar method to that used by Brennan and Fox (1995), Brennan and Quade (2004), and Lantican et al. (2016) was used to estimate the economic impact of the CAIGE bread wheat germplasm in Australia. The approach relied on the assumption that the percentage of CAIGE material in a cultivar reflects the same percentage yield advantage. Traits for water use efficiency and disease resistance, for example, may produce a different yield benefit in a different season but were not considered in the analysis as such data, including the contribution of traits from CAIGE material to yield, were unavailable. Nevertheless, the approach was built upon previous published estimations of economic impact. Net benefits of wheat genetic materials imported to Australia from the CAIGE program between 2005 and 2019 were estimated. The first 5 years of the program were assumed to be breeding years. The benefit period to Australian producers and consumers of wheat was set at 2010/2011 to 2034/2035 to account for the development lag between material identification and commercial release of new cultivars.

#### 2.3.1 Data resources

**Figure 2:**
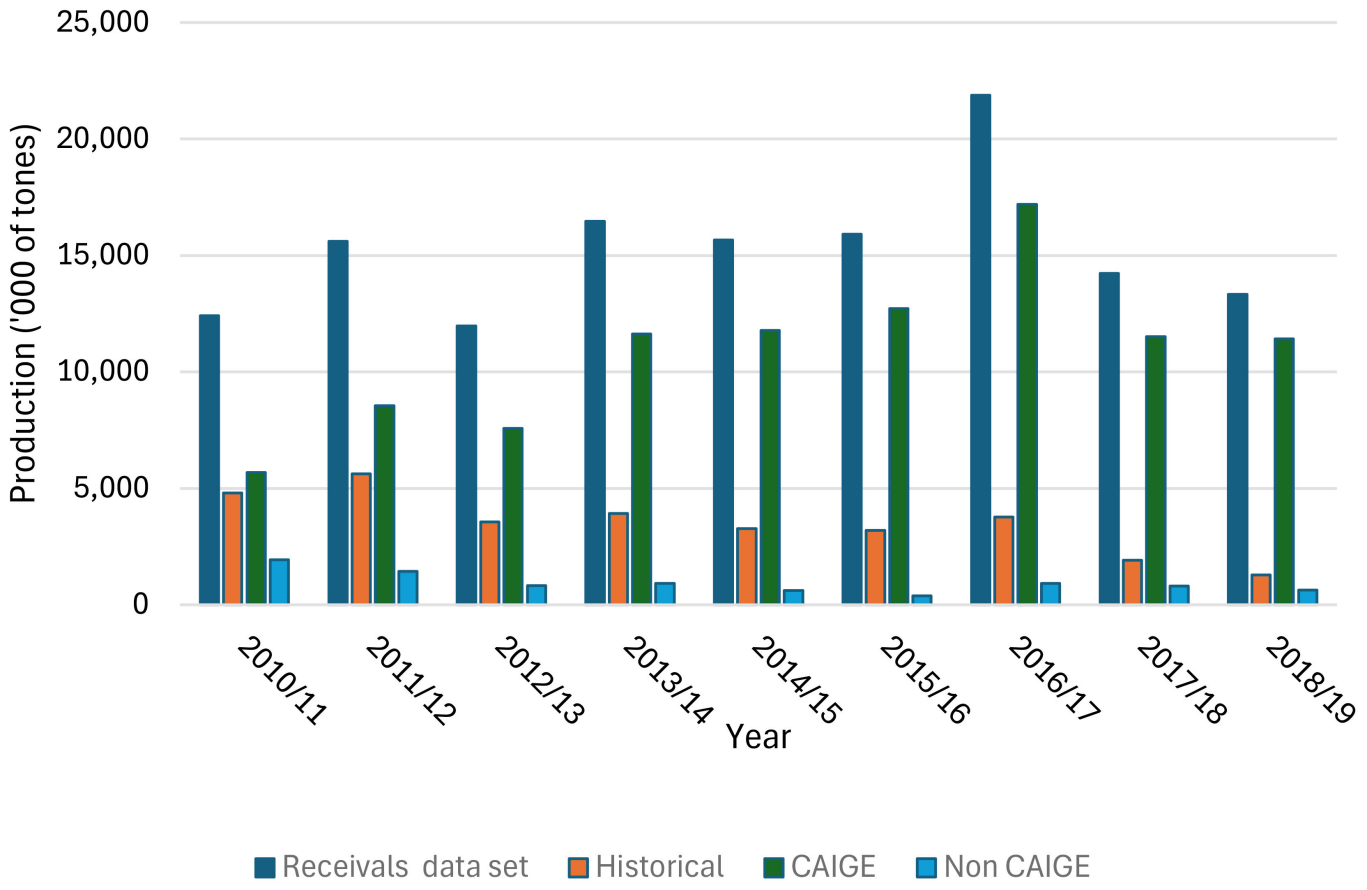
Summary of wheat grain (in ‘000 of tonnes) produced nationally including total grain receivals, and receivals of historic, and CAIGE- and Non-CAIGE–derived cultivars.

The analysis focussed on CAIGE materials imported from 2005, which were incorporated into cultivars released in Australia between 2010–2011 and 2018–2019. The GRDC manages a dataset that contains cultivars received by Australian grain handlers (grain accumulators), which account for approximately 82% of Australia’s annual wheat production. The remaining 18% represents feed grain lines that were used on farm. The dataset included tonnes, grades, and region of production for each cultivar by year from 2010/2011 to 2018/2019. The pedigrees of cultivars in the Grain Handler dataset for this period were analysed to derive the percentage of material sourced from the CAIGE program using published pedigrees and information directly sourced from wheat breeders. Some cultivars in the dataset were up to 25 years old. Cultivars that were commercially released prior to 2010/2011 were removed. These were labelled as “Historic” in **Figure 2**.

A summary of the GRDC Grain Handler set is provided in **Figure 2**. It shows the breakdown of tonnes derived from CAIGE and Non-CAIGE lines. Non-CAIGE and CAIGE data were partitioned into two subsets using the following criteria:

1) Non-CAIGE = cultivars released with <1% material in pedigree derived from CAIGE program.

2) CAIGE = cultivars released with >1% material in pedigree derived from CAIGE program.

#### 2.3.2 Estimation of benefits

Pedigrees of commercial wheat cultivars released in Australia were analysed to calculate the percentage of CAIGE material in each cultivar. The percentage of CAIGE-derived material in a cultivar was then multiplied by its production tonnes each year in the grain handler dataset. That value was then multiplied by 1.4% to represent the annual average yield advantage of CAIGE versus Non-CAIGE cultivars. Favourable germplasm is generally carried forward into new cultivars for at least three generations (Pardey, 2011). The assumption used in this analysis was that genes from this germplasm would be present in new cultivars for a period up to 25 years. We therefore projected yield gains to peak at year 10 and then decline to zero by 2034–2035. The projection was calculated from the nine seasons of available data and then extrapolated for a period of 16 years.

Annual prices paid to growers were sourced from Australian Bureau of Agricultural and Resource Economics and Sciences (ABARES) (2021). These prices were inflated to 2020 values (real prices) using the December Consumer Price index (CPI) (ABS, 2021). Kingwell (2017) shows that the real price of wheat in Australia decreased by 2.1% from 1973–1974 to 2015–2016.

Next, the long-term demand elasticity was calculated for Australian wheat produced between 2001 and 2020 using the average of the first 10 years versus the second 10 years as the change period. The elasticity was −39.93 and represents the percentage change in tonnes for a 1% change in prices. The reciprocal of the price elasticity is the price flexibility. This measures the change in price for a 1% change in tonnes, which was −0.025. That is, for each additional 1% of wheat supplied, the price to growers fell by 0.025%. Past and future prices were decreased by the annual percent change in tonnes above the mean tonnage for the analysis period.

The tonnes of production attributed to CAIGE with the deductions described above were multiplied by the price adjusted for CPI and the price flexibility to derive a benefit value.

Wheat breeders earn a 3% end point royalty on new wheat lines in Australia, which is charged on a value basis, and this value was deducted from the benefit value. Benefits in the period beyond 2020 to 2035 were discounted using a 5% discount rate.

#### 2.3.3 Historical investment in CAIGE

GRDC and the Australian Government through the Australian Centre for International Agricultural Research (ACIAR) invest in various wheat breeding programs and activities at the CGIAR centres. Combined funding of $13.6 million was received by the CGIAR centres between 2005 and 2020 for wheat breeding related research. Of this, $5.57 million or 45.5% was invested by GRDC in CIMMYT, ICARDA, and university research and delivery programs. Some of the ACIAR funding was specifically used in foreign research programs although some benefits may have flowed back to Australia. ACIAR (2021) lists several research programs that delivered benefits to farmers in India, Pakistan, Bangladesh, Afghanistan, and other countries. Potential benefits from these projects were excluded from this analysis.

## 3 Results

### 3.1 Genetic impact of the CAIGE program

The CAIGE program significantly improved the acquisition of germplasm adapted to Australian conditions, confirmed yield and disease responses in Australia, and shared this information in a timely way through the CAIGE website to facilitate germplasm uptake and use by Australian wheat breeders and researchers.

Analysis of data generated in the period 2017–2020 highlighted the value of the CAIGE program for identifying new germplasm for Australian wheat breeding. The environment mean yield for this period ranged from 0.62 t/ha to 6.59 t/ha, which is representative of Australian wheat production environments. The reliability, equivalent to the line mean broad sense heritability for a balanced design, ranged from 0.35 to 0.90 (**Table 1**). The mean total genetic variance was 0.178, ranging from 0.014 at Corrigin, WA, in 2019 to 0.975 at Spring Ridge, NSW, in 2020 (**Appendix Table 1**).

Between-year genotype connectivity was very low (minimum of 9 and maximum of 21), except for Spring Ridge 2020 because the 2019 genotype list was re-evaluated alongside the 2020. Parental connectivity across years was reasonable with a minimum number of 25 parents in common between environments (data not shown).

Initial modelling of the dataset commenced using an independent genetic variance model, where no relationship between genotypes or environments was considered and culminated in a final model where the additive and non-additive GE variance–covariance matrices were fitted with factor analytic models of order ka = 4 and ke = 2, respectively. This model had the maximum likelihood, the lowest AIC and the largest % total genetic variance accounted for (%vaf) with 81%. The mean %vaf by the individual additive factors was 46%, 17%, 14%, and 9%.

**Figure 3:**
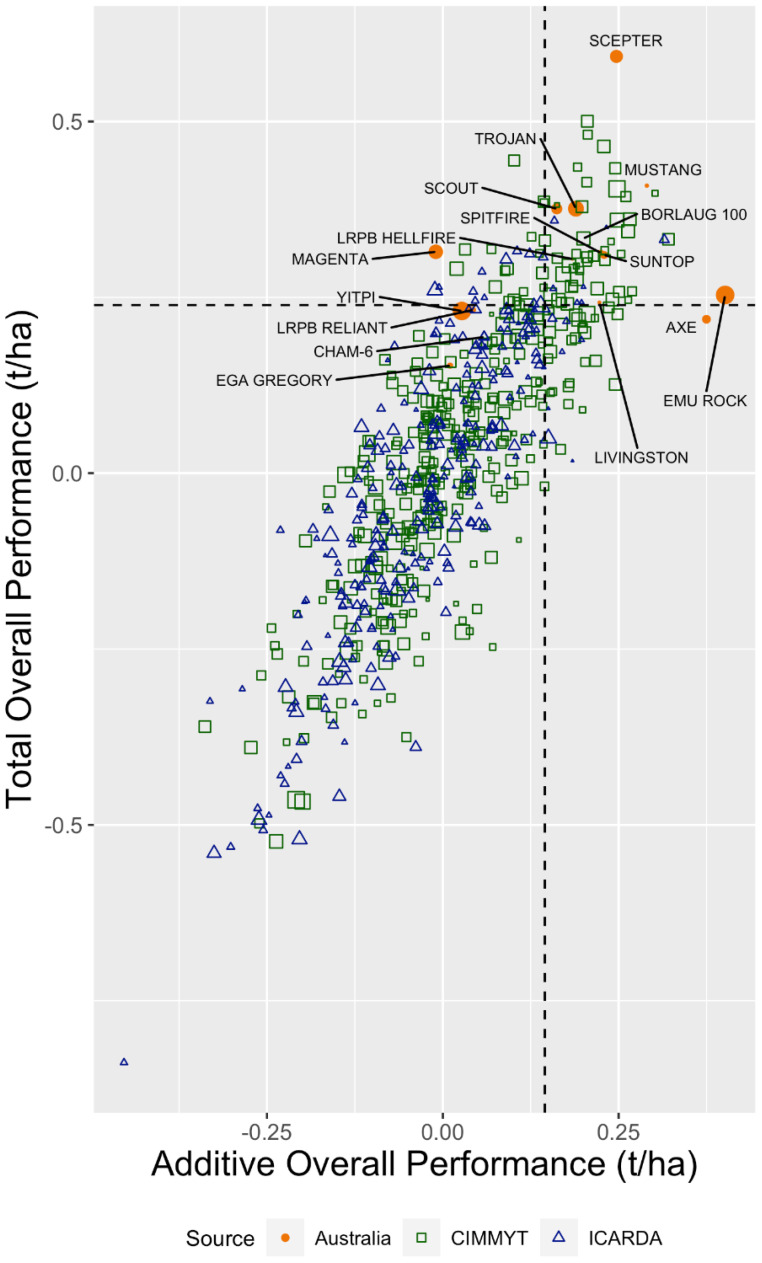
Overall performance for total and additive effects for the Australian, CIMMYT, and ICARDA 2020 entries in the 2017–2020 dataset. The dashed horizontal and vertical lines represent the top 10% cutoff values for the total and additive overall performance, respectively. The Australian, CIMMYT, and ICARDA check cultivars are labelled. The size of the point indicates the additive stability (RMSD) of the genotype with a smaller point indicating high stability.

**Figure 3** shows the genotypes evaluated in 2020. To select breeding lines for direct release, the total OP (vertical axis) would be used; however, to select lines for use as parents, the additive OP is more relevant.

**Figure 4:**
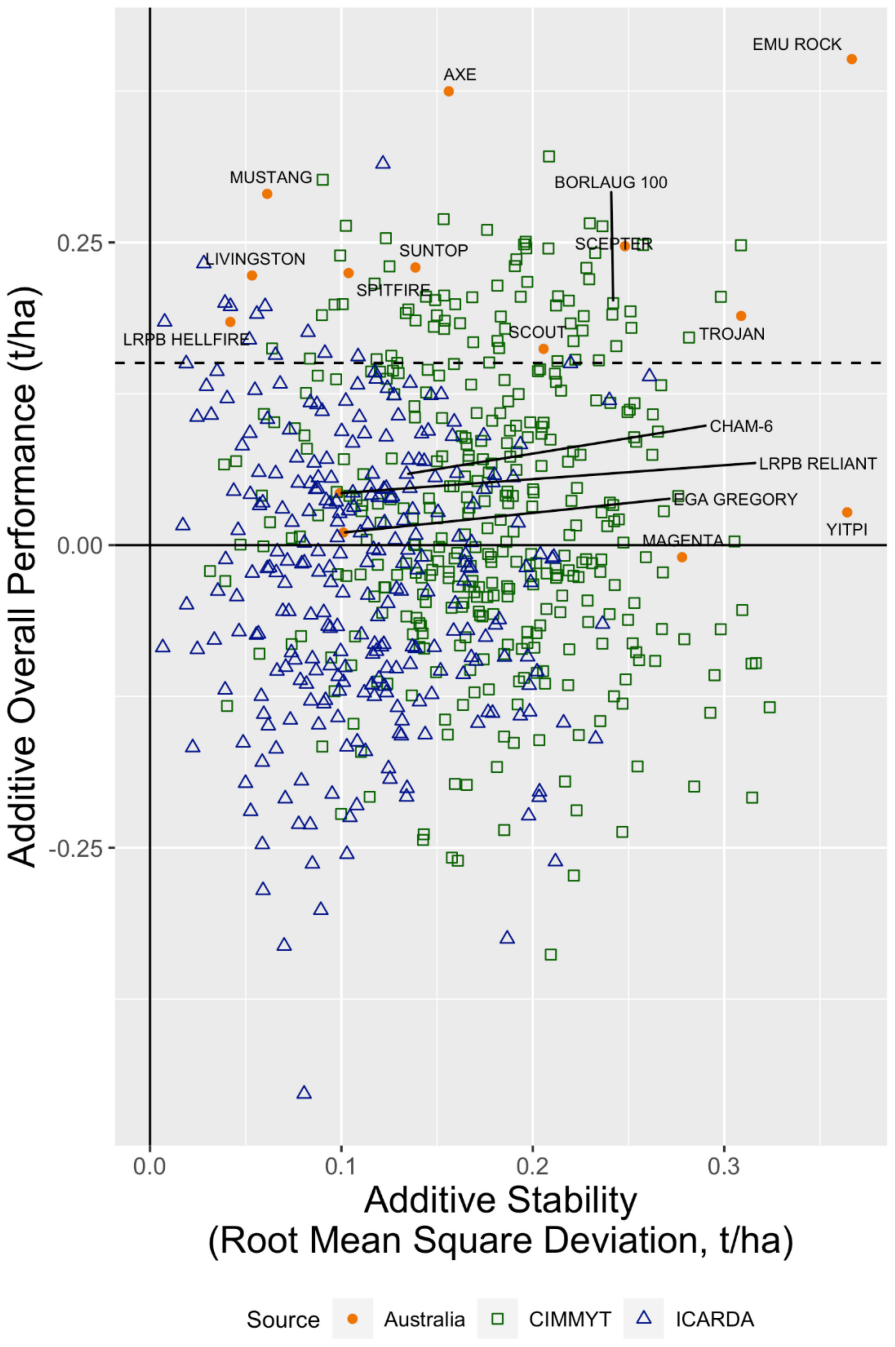
Overall performance versus stability for the additive genetic effects of the Australian, CIMMYT, and ICARDA lines evaluated in 2020. The Australian, CIMMYT, and ICARDA check cultivars are labelled. The horizontal dashed line indicates the top 10% cutoff for additive overall performance.

OP and RMSD (stability) for the additive effects were used to summarise genotype performance (**Figure 4**). The first factor for the additive effects, on which OP and RMSD are based, accounted for 46% of the additive genetic variance. Genotypes in the top left-hand corner have good additive OP and maximum stability (lower RMSD) across environments. Some imported lines outperformed the Australian cultivars, demonstrating potential value as parents. There was a considerable overlap between CIMMYT and ICARDA genotypes with respect to both OP and stability.

**Figure 5:**
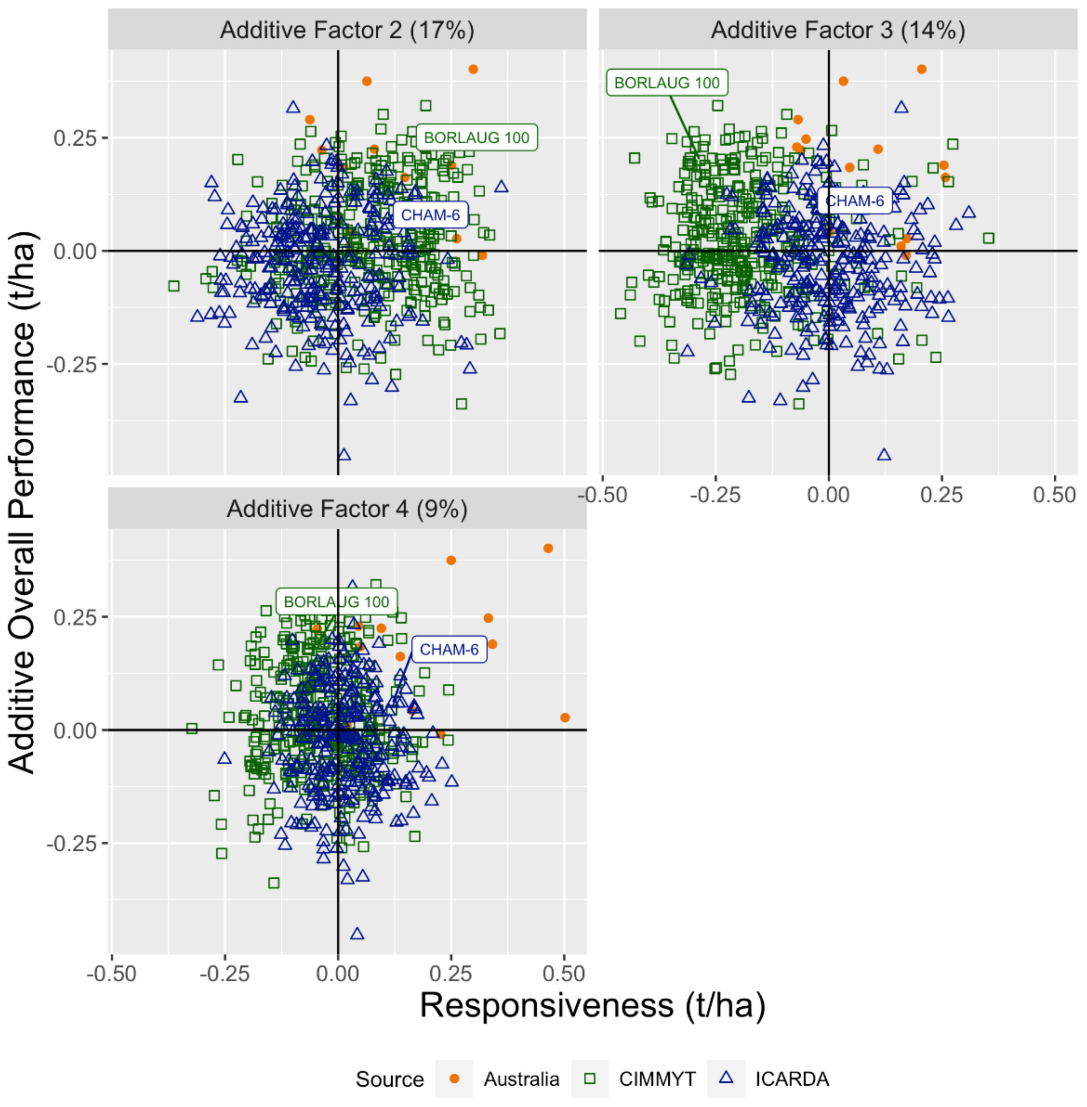
Overall performance versus responsiveness for the Australian, CIMMYT, and ICARDA 2020 entries for factors 2 to 4 fitted to the additive genetic variance–covariance matrix. The CIMMYT and the ICARDA check cultivars are labelled.

The FAST responsiveness statistics described the remaining 40% of the 86% estimated additive genetic variance. **Figure 5** presents the additive OP compared to responsiveness for factors 2 to 4, each in a separate panel. The contrasting performance of the check genotypes can help identify the underlying environmental covariates driving these factors. For example, for factor 2, the responsiveness of the CIMMYT check (BORLAUG 100) and the ICARDA check (CHAM-6) is similar, whereas, in factors 3 to 4, these two genotypes respond differently.

The number of genotypes by germplasm source present in the top 10% based on additive and total OP in each year cohort is given in **Table 2**. The numbers were summarised from the additive and total OP calculated for each genotype. Genotypes from both CIMMYT and ICARDA appeared in the top 10% additive and/or total effects which, combined with other traits, may be of interest to collaborating breeders.

**Table 2:** Number of genotypes by year and germplasm source evaluated in multi-environment trials appearing in the top 10% for additive and total overall performance.

Year	Australian	CIMMYT	ICARDA
No. of genotypes			
2017	12	112	111
2018	14	164	135
2019	12	178	105
2020	15	373	271
Total	53	827	622
No. of genotypes in top 10% additive			
2017	7	6	10
2018	10	13	6
2019	7	17	3
2020	10	48	7
Total	34	84	26
No. of genotypes in top 10% total			
2017	9	6	8
2018	7	17	5
2019	7	16	4
2020	9	46	10
Total	32	85	27

The international germplasm provided a significant source of resistance to the three rust diseases with most genotypes recording resistant to moderately resistant reactions in each year (**Table 3**). The international genotypes were also sources of resistance to many foliar and soil-borne pathogens. A small number of sources of resistance were identified for *S. tritici*, and many were identified for *S. nodorum*. Furthermore, several genotypes resistant to *S. nodorum* resistance were also resistant to tan spot (*Pyrenophora tritici*). Septoria nodorum blotch caused by *Parastagonospora nordorum* was also assessed by testing host sensitivity to one of the three fungal effectors: ToxA, Tox1, and Tox3. A high number of insensitive lines to ToxA were found in the CAIGE materials, which can be used to enhance disease resistance potential of wheat. Seedling resistance to the stubble borne disease tan spot was common among genotypes evaluated in all years; however, fewer genotypes were resistant at the adult plant stage. A small number of CAIGE genotypes also showed partial resistance to the tractable crown rot disease.

**Table 3:** Percentage of genotypes that were rated as resistant or moderately resistant based on pathological screening of CAIGE bread wheat materials across Australia, 2017–2020.

Disease	Percentage of genotypes rated R or MR^2^
Stem rust	93
Leaf rust	81
Stripe rust	73
Septoria tritici (adult plant)	1
Septoria nodorum blotch (adult plant)	60
Yellow spot (seedling)	19
Yellow spot (adult plant)	32
ToxA^1^	82
Crown rot	14

^1^ indicates insensitivity. ^2^R is resistant, and MR is moderately resistant.

Overall, high-yielding and disease-resistant germplasm that was competitive with and potentially different from the Australian check cultivars was identified.

### 3.2 Economic impact of the CAIGE program

The estimated economic impact of CAIGE bread wheat germplasm in Australia was significant. Statistics for the Non-CAIGE and CAIGE subsets of varieties are shown in **Table 4**. These data show that the tonnes of Non-CAIGE lines decreased from 1.9 million tonnes in 2010–2011 to 0.6 million tonnes. However, during the same period, the tonnes associated with CAIGE lines increased from 5.6 million tonnes to 11.4 million tonnes in 2018–2019. The total number of Non-CAIGE varieties increased from 9 to 12 over the data period relative to an increase of 38 to 53 lines for the CAIGE set.

**Table 4:** Summary of the grain production of CAIGE and Non-CAIGE cultivars in Australia by year including number of cultivars, average tonnes, and standard deviation from the mean.

	2010/2011	2011/2012	2012/2013	2013/2014	2014/2015	2015/2016	2016/2017	2017/2018	2018/2019
CAIGE									
Number of cultivars	38	38	44	46	48	54	59	60	53
Average Tonnes	149,341	225,039	172,178	252,764	245,608	235,497	291,275	191,836	215,318
STDEV.S	342,175	590,198	632,308	1,169,040	1,166,856	1,137,839	1,337,456	763,462	885,385
Non-CAIGE									
Number of cultivars	9	10	10	11	12	10	12	13	12
Average Tonnes	215,345	142,925	82,700	82,729	50,279	39,148	77,004	61,843	53,136
STDEV.S	418,845	258,701	130,071	122,203	72,754	34,014	150,431	177,817	151,213

Australian wheat production for the period 1988–2089 to 2021–2022 increased by 175,000 tonnes per year (ABARES, 2021). Droughts were experienced in each decade; however, productivity in the most recent decade was, on average, higher than that in the previous two decades.

Average Australian wheat yields were higher in the previous decade than earlier decades (ABARES, 2020). While some of this change was due to changing farming practices including no-till seeding, controlled traffic, stubble retention, and chemical weed control, many of these practices were widely adopted over two decades ago, implying that genetic gain has been mostly responsible for the most recent yield increases.

GRDC Grain Handler data showed that 71% of Australian wheat production is derived from CAIGE-related cultivars relative to 29% from Non-CAIGE cultivars. Finally, national data showed that yields and production were at least 200 kg higher in the most recent decade relative to the previous two decades.

The results of the cost-benefit analysis are shown in **Table 5**. The present value of the investment and benefits are shown for the proportion of the CGIAR wheat research that was Australian-funded. The GRDC and Partner contributions and benefits are shown in the second column. The attribution to GRDC and its partners is split by investment proportion (45.5%) rather than ascertain a split by project outcome. The net present value or the sum of benefits minus the sum of investments reflects the value of the investment and benefits in 2020 dollars. Similarly, the benefit-cost ratio or sum of benefits divided by investments showed that, for every dollar invested, a further $20 was generated in benefits. Finally, the internal rate of return was 163%, and the modified rate was 18%.

**Table 5:** Benefit-cost analysis of CAIGE materials including total Australian investment in the Consultative Group on International Agricultural Research (CGIAR) for the period and that attributed to Grains Research and Development Corporation (GRDC) support.

	Total Australian investment in CGIAR	GRDC component of investment (45.5%)
Sum of investment	14,194,045	6,458,290
Sum of benefits	286,444,855	130,332,409
Net present value	272,250,811	123,874,119
Benefit-cost ratio	20.18	9.18
Internal rate of return	163%	74%
Modified rate of return	18%	8%

Sum of investments and benefits are the present values in 2020 at CPI inflation rates for past investments and benefits and 5% discount rate for future investment and benefits. Modified rate of return used 3% capital and 5% return rates.

The CAIGE materials clearly contributed to yield improvement and provided a significant economic benefit over the period of the study.

## 4 Discussion

The CAIGE program has significantly improved the evaluation, access to information, and local uptake of bread wheat germplasm from the CGIAR centres while providing a reciprocal benefit to international agriculture through timely access to data on a continental scale and the exchange of Australian cultivars and their use to develop new germplasm for the developing world. A number of cultivars carrying Australian parentage have been released in developing countries in recent years (wheatatlas.org/varieties).

### 4.1 Genetic impact of the CAIGE program

Analysis of the CAIGE data for the period 2017–2020 provided a snapshot of the adaptation and trait values of bread wheat germplasm selected and imported from the CGIAR centres. In most environments, subsets of CAIGE genotypes performed well compared to the Australian check cultivars, and many carried high levels of disease resistance. Some imported lines with good additive OP and maximum stability were superior to the Australian cultivars, demonstrating their potential value as parents. There was considerable overlap between CIMMYT and ICARDA genotypes with respect to both OP and stability. These results justify the acquisition of germplasm from both international centres given that the materials are genetically distinct based on pedigree. Although the frequency of CAIGE materials resistant to soil-borne diseases was low compared to that of most foliar pathogens, the resistant sources identified do provide wheat breeders with potentially new diversity that can be confirmed following genetic analysis. The collection of data and dissemination of information via the CAIGE website and other meetings and resources has provided a solid basis for the selection of materials for direct release and/or use as parents in the breeding process. This will only be enhanced by the provision of genotyping information on all imported materials from 2023 onwards. Clearly, wheat breeders seek unique sources of resistance and yield potential, and the genetic data will assist with the selection of genotypes potentially carrying new diversity for key traits.

### 4.2 Economic impact of the CAIGE program

The estimation of economic impact indicated a significant return on the research investment in CAIGE. Brennan and Quade (2004) estimated annual returns of approximately $30 million per annum relative to $11.4 million per annum in this analysis. Brennan and Quade did not use pedigree percentage attributed to CGIAR material in their analysis, and, therefore, considering a similar number of cultivars, their benefit value would have been higher per cultivar. They mitigated this to some extent, over time, by reducing the benefit value of second-generation cultivars containing CAIGE materials.

Their analysis ran for a period of 48 years including extrapolation to 19 future years, whereas this analysis ran across 30 years including a 5-year breeding phase with no benefits and 16 future years. They had to account for the ramp up of breeding materials but did not have to account for a large proportion of preexisting cultivars in production. Sixty percent of the tonnes produced in 2010/2011 were derived from CAIGE-derived cultivars. At the end of our data period (2018/2019), 10% of annual production in Australia was still attributed to varieties included in the 2004 Brennan and Quade analysis. The future adoption period estimated by Brennan and Quade appears to have been too conservative and may well be too conservative in our case.

In addition to the direct yield benefit, there are other traits in the CAIGE-derived varieties that save costs associated with crop production. These include increased disease and pest resistance (a significant percentage of the CAIGE materials carried multiple diseases resistances), which enable grain growers to apply fewer chemicals. These benefits could be substantial as the CAIGE program has access to CGIAR global disease hotspots; thus, the imported materials frequently have resistance to both local and international biotypes, including the yet to be introduced stem rust strain Ug99 (Singh et al., 2011). This global screening reduces risks associated with disease introductions or pathogen mutations (Smale and Singh, 1998). However, these cost savings are not accounted for in the economic analysis. In future, it would be useful to record the dominant traits of the CAIGE genetic materials, which would enable the analysis to be more precise. Meng and Brennan (2009) provide a suitable method of analysis if the data were available.

While the benefits of investment in CAIGE are significant, it also worth considering the counterfactual case that assumes no Australian (GRDC and Government and Research partners) investment in the CAIGE program. In this case, Australian wheat breeders would have been limited to smaller sets of genetic materials suited to Australian conditions developed by the CGIAR and no supporting data. Australia’s share of the total CGIAR wheat budget was approximately 6% (data from FPU 2011 to 2020). Other countries and research partners may have continued to test wheat lines. These lines may or may not have been suited to Australia’s environment. Therefore, it was assumed that genetic material would still have been available to breeders although at a higher cost though an alternative licence fee structure and without supporting information. The yield gains and tonnages of Non-CAIGE lines was used to represent this counterfactual case, and it was clear that rates of genetic gain for yield would still have been significant but lower.

Many studies of yield gains such as that presented here include an analysis of producer and consumer welfare or surplus that accrues from the investments undertaken. These measures were first promoted by Dupuit in 1844 as cited by Currie et al. (1971). Consumer surplus is a measure of utility derived from the purchase and consumption of goods at a particular price point. One issue with the surplus method is that wheat is an input into further manufacturing processes and a range of goods are produced at different price points. Therefore, a sum of the utility of these various goods is required. Consumer surplus is also dependant on changes in population, incomes, prices, and substitution of other commodities. Foreign production and consumption play a large role in the value of wheat to Australia and its overseas buyers. Given that we have not accounted for these variables in this analysis, it was not prudent to estimate or report consumer surplus measures.

Similarly, producer surplus is a function of change in the price, cost, and relative profitability of one crop against another. Non-monetary benefits including disease management are often neglected. Variable crop production costs increased in Australia by $19/ha over the analysis period; however, on a dollar-per-tonne basis, the production costs decreased by $38. A proportion of these costs were due to the nutrient requirements of the crop to produce a higher yield. The yields recorded were also a function of annual rainfall, which is hidden in cost measures. Benefit attribution from the cost reduction is, therefore, not a simple calculation. We do not consider welfare measures to be suitable to report here as a measure of impact.

The analysis presented here focussed on milling wheats only (excluding durum and barley, both more recent CAIGE additions) and the benefits of CAIGE program participation to Australian producers and consumers of wheat. International consumers were not considered nor wheat breeding benefits flowing to other countries in this analysis.

## 5 Conclusion

The CAIGE model provides a platform for deriving the best possible benefit from the international investment in public wheat breeding for the benefit of Australian grain growers. The CAIGE concept can serve as a model for other countries, with adjustments for local conditions and culture. While the CGIAR germplasm remains a significant source of publicly available genetic diversity accessed by all Australian bread wheat breeding programs, there are also reciprocal benefits attributable to CAIGE as Australian materials, and data are also accessed and used by CGIAR breeders to improve the international germplasm.

## Data availability statement

The datasets presented in this study can be found in online repositories. The names of the repository/repositories and accession number(s) can be found below: http://www.caigeproject.org.au.

## Author contributions

RT: Conceptualization, Funding acquisition, Investigation, Methodology, Project administration, Resources, Supervision, Writing – original draft. JN: Data curation, Investigation, Methodology, Writing – review & editing. AS: Data curation, Investigation, Methodology, Validation, Writing – review & editing. RS: Investigation, Validation, Writing – review & editing. WT: Investigation, Writing – review & editing. VG: Investigation, Writing – review & editing. LC: Investigation, Writing – review & editing. BC: Formal analysis, Writing – review & editing. LM: Formal Analysis, Writing – review & editing. MD: Conceptualization, Writing – review & editing. SM: Investigation, Writing – review & editing. TF: Formal Analysis, Writing – review & editing. RW: Data curation, Writing – review & editing. KM: Formal analysis, Investigation, Writing – review & editing.
