# Supplementary material for: The CIMMYT Australia ICARDA Germplasm Evaluation concept: a model for international cooperation and impact
Source: Front Plant Sci. 2024 Jul 30;15:1435837. doi: 10.3389/fpls.2024.1435837 (PMC11319148; doi:10.3389/fpls.2024.1435837)
Supplement: Supplementary file 1 [file Table_1.pdf]

Appendix Table 1. Trial locations, years sown and associated statistics for the CAIGE bread wheat data set 2017 – 2020.

State	Nearest town	GPS location	2017	2018	2019	2020	Number of Plot	Number of Genotypes	Partials replication (%)	Trial mean yield (t/ha)	Reliability	Genetic Variance	Additive Variance (%)
Queensland (QLD)	CONDAMINE	27°S 150°E				✓	288	252	14	4.1	0.61	0.11	30
	GATTON	28°S 152°E				✓	288	244	18	2.8	0.63	0.24	0
New South Wales (NSW)	JUNEE	35°S 148°E	✓	✓		✓	240-420	208-353	14-67	1.6-4.3	0.82-0.90	0.03-0.37	22-55
	NARRABRI	30°S 150°E	✓	✓	✓	✓	420-624	234-501	24-100	3.8-5.7	0.66-0.88	0.15-0.24	14-53
	NORTH STAR	29°S 150°E	✓		✓	✓	288-360	180-317	5-61	0.8-4.2	0.48-0.77	0.02-0.16	39-55
	EDGEROI	30°S 150°E		✓			240	197	19	3.3	0.67	0.07	61
	SPRING RIDGE*	31°S 150°E				✓	288	264	7	4.9	0.79	0.97	23
	SPRING RIDGE*	31°S 150°E				✓	288	232	19	4.6	0.79	0.97	23
Victoria (VIC)	HORSHAM	37°S 142°E	✓				288	190	52	6.6	0.78	0.34	53
	SWAN HILL	35°S 143°E			✓		288	233	19	0.6	0.79	0.06	49
	LALBERT	36°S 143°E				✓	288	264	9	2.9	0.85	0.16	30
	LONGERENONG	37°S, 142°E				✓	288	257	12	3.2	0.35	0.12	56
South Australia (SA)	BALAKLAVA	34°S 138E	✓			✓	336-420	213-400	5-56	2.3-2.5	0.56-0.81	0.03-0.10	29-39
	ROSEWORTHY	35°S 139°E	✓	✓		✓	240-576	206-501	14-64	2.7-5.1	0.71-0.89	0.12-0.46	22-49
Western Australia (WA)	TOODYAY	32°S 116°E	✓	✓	✓		240-312	201-221	17-55	2.5-4.5	0.47-0.65	0.06-0.19	31-54
	DANDARAGAN	31°S 116°E		✓	✓		240-288	194-221	20-21	2.3-3.7	0.53-0.81	0.04-0.27	8-49
	CORRIGIN	32°S 118°E			✓	✓	288	232-245	18-24	0.9-1.2	0.65-0.71	0.01-0.07	33-57
	GOOMALLING	31°S 117°E			✓	✓	288-420	245-319	18-32	2.0-2.3	0.52-0.56	0.02-0.04	33-42
	MINGENEW	29S 115°E				✓	288	271	6	4.0	0.67	0.23	34
	YORK	32°S 117°E				✓	288	269	7	2.3	0.63	0.05	45

*Two trials grown at Spring Ridge in 2020.
